# Applying a Novel Combination of Techniques to Develop a Predictive Model for Diabetes Complications

**DOI:** 10.1371/journal.pone.0121569

**Published:** 2015-04-22

**Authors:** Mohsen Sangi, Khin Than Win, Farid Shirvani, Mohammad-Reza Namazi-Rad, Nagesh Shukla

**Affiliations:** 1 SMART Infrastructure Facility, University of Wollongong, Wollongong, New South Wales 2522, Australia; 2 School of Information Systems & Technology, Wollongong University of Wollongong, New South Wales 2522, Australia; The State University of New York at Stony Brook, UNITED STATES

## Abstract

Among the many related issues of diabetes management, its complications constitute the main part of the heavy burden of this disease. The aim of this paper is to develop a risk advisor model to predict the chances of diabetes complications according to the changes in risk factors. As the starting point, an inclusive list of (k) diabetes complications and (n) their correlated predisposing factors are derived from the existing endocrinology text books. A type of data meta-analysis has been done to extract and combine the numeric value of the relationships between these two. The whole n (risk factors) - k (complications) model was broken down into k different (n-1) relationships and these (n-1) dependencies were broken into n (1-1) models. Applying regression analysis (seven patterns) and artificial neural networks (ANN), we created models to show the (1-1) correspondence between factors and complications. Then all 1-1 models related to an individual complication were integrated using the naïve Bayes theorem. Finally, a Bayesian belief network was developed to show the influence of all risk factors and complications on each other. We assessed the predictive power of the 1-1 models by R^2^, F-ratio and adjusted R^2^ equations; sensitivity, specificity and positive predictive value were calculated to evaluate the final model using real patient data. The results suggest that the best fitted regression models outperform the predictive ability of an ANN model, as well as six other regression patterns for all 1-1 models.

## Introduction

Diabetes is spreading all around the world as an unprecedented epidemic: in 2011, 366 million people had diabetes and by 2030 this will have risen to 552 million (8.3% compared to 9.9% of the adult population, respectively) [1].

Among many related issues of diabetes management, its complications constitute the main part of the heavy burden of this disease. They represent around 60% of direct and almost 80–90% of the indirect related costs [2]. These complications can be prevented if the treatment starts before the development of non-reversible clinical symptoms. This means that predicting them is essential in order to intervene successfully. E.g. early detection and proper treatment of diabetes can prevent up to 90% of blindness, at least 50% of kidney failure and nearly 80% of amputations [3].

Diabetes and its complications have been well studied over the past 20 years and a great deal of energy has been put into guidelines, best practice, optimization of care and other management methods to improve outcomes. However, compliance with the recommended preventive care for diabetic patients has been low [4, 5]. The discrepancy between what is known and what is done in diabetes care indicates that better knowledge management is necessary in order to improve results through sharing and making use of the information. Several studies have documented a significant gap between actual clinical practice and optimal patient care. For example, in a systematic review of the quality of health care in the United States, Schuster et al. found that only about 60% of patients received recommended care for chronic conditions [6]. Because of this, computer-based clinical reminder systems are often seen as an effective strategy to promote preventive procedures generally and in diabetes management, in particular [7–10]. These facts justify the development of an advisory tool to help in the early and prompt detection of diabetes complications.

This paper presents the model development for predicting the complications of diabetes by systemically assessing the current literature on clinical trials. We gathered the results of the previously conducted surveys. Each survey has studied a number of patients during a period of time to investigate the quantitative relationships between a series of diabetic risk factors and complications. Using the results of these studies enabled us to build a model based on the information from more than 450,000 patient/years. In the end, the real data of 84 diabetic patients from one of the largest Australian longitudinal population-based studies (AusDiab [26]) has been used in order to externally validate the final model.

## Background

Since diabetes is a multi-factorial disease which is regulated by multiple genes and a number of environmental factors, there is a need for predicting complications in order to reduce the economic and social burden of the disease [11].

### Computerized decision tools in diabetes

Apart from the broad categories of the use of medical informatics in the health care area, its main applications with regard to diabetes have been grouped into three major categories: the prompting of diabetes care, insulin dose adjustment and patient education [9, 12]. There have been expert systems and decision support systems (DSS) to advise on patient management, computer algorithms and artificial intelligence to regulate insulin dosage, and a range of mathematical models as well as approaches drawing upon optimal or adaptive control [13–16] in order to manage the disease more effectively and decrease the burden of its complications.

### Problem with current decision tools

There are limitations, however, which are common to all of these models. Firstly, it would not be possible to add any new risk factor to the model which was not considered at the time of the initial study. In addition, all models are created based on a specific population that makes it hard to generalize to others. The last but not the least limitation is, most of the existing systems use different risk factors to predict only a single complication, not all complications.

The literature shows no decision tools to individually predict the absolute risk of all diabetes complications using individual patient data. The main reasons for failure and the difficulty of designing a beneficial decision system are inadequate usable data at hand and an over-emphasis on technology [17]. In order to implement an empirical tool to predict absolute risk of diabetes complications, these two obstacles must be overcome. Sufficient reliable data must be gathered and correctly structured methods must be applied.

This study was approved by the Human Research Ethics Committee at the University of Wollongong.

## Methodology

The overall goal of this paper is to design a predictive risk advisor by modelling the relationships between diabetic predisposing factors and their related complications. Because creating a final n-k model for all risk factors and complications is very complicated, diabetic retinopathy—and this includes any kind of diabetic retinopathy (DR), non-proliferative diabetic retinopathy (NPDR) and proliferative diabetic retinopathy (PDR)—as well as diabetic nephropathy, which includes both microalbuminuria and macroalbuminuria, are selected as diabetic complications. Among all the related predisposing factors for these complications, duration of disease and HbA1c for both groups, and albumin exertion rate (AER) and blood pressure control (BP) for each group, respectively, are investigated. As a prerequisite, the relationship between selected risk factors and complications needs to be extracted through a secondary research approach and then, the data is presented in table format.

**Fig 1:**
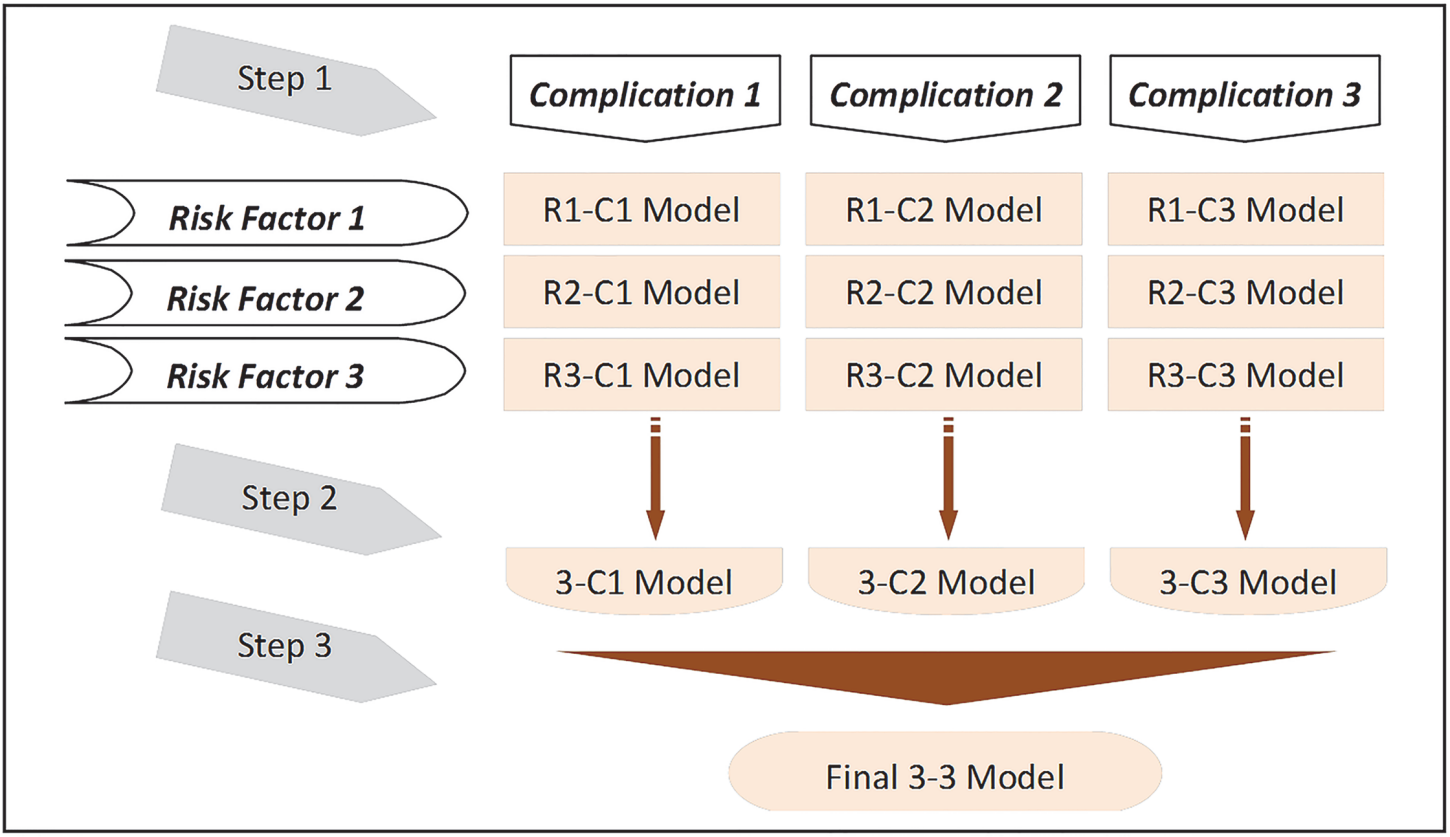
A perspective of system development steps.

Another specific goal of this research is to design an innovative procedure to break down the final model into simpler steps, including modelling the relation between one risk factor and one complication (step 1), modelling the relation between n risk factors and one complication (step 2), modelling the relation between n risk factors and k complications (step 3) and then identifying the most reasonable methods and empirical techniques for modelling each step. Fig 1 presents an outline of these steps.

This model uses individual patients’ current and previous data as its input and estimates the relative risk of each diabetic complication as its output. Having created the final model, physicians and other healthcare workers can thus predict the probability of complications by observing patients’ individual data, and this will result in better management of diabetic complications.

Classification is a form of data analysis technique that can be used to extract models describing important data classes and predict future data trends. There are several popular techniques that dominate tools for classification and prediction including neural networks (NNs), naïve Bayes, Bayesian networks, decision trees (C4.5), association rules and support vector machines (SVMs). Some of these techniques may be used for both prediction and classification, while others have been used specifically for classification. We have used some of the abovementioned methods for classification and predictions. There have been numerous comparisons of the different prediction and classification methods but no single method has been found to be superior to all others for all data sets [18, 19]. Issues such as accuracy, training time, robustness, interpretability and scalability must be considered and can involve tradeoffs, further complicating the quest for an overall superior method. Empirical studies show that the accuracy of many algorithms is so similar that their differences are statistically insignificant [20–22].

Least square regression and artificial neural networks have been chosen for the first step. The naïve Bayes is selected as a classifier for the second step as it can deal with the independent factors. The naïve Bayesian classifier assumes class conditional independence. When the assumption holds true, it is the most accurate compared with all other classifiers. The Bayesian network has an appealing, transparent and intuitively clear structure which can be visualised graphically. In addition, because all relations between variables are described by the rules of probability, there are no assumptions made by the method. A Bayesian network is, therefore, used to illustrate the relation among all the risk factors and diabetic complications. Finally, the accuracy of the model is evaluated by applying real patients’ data from the Australian diabetes, obesity and lifestyle study (AusDiab), the largest Australian longitudinal population-based study [23].

### Data preparation

There are two central tasks for data preparation in this research.

#### Data gathering

First of all, a knowledge base is created, which uses some knowledge representation structures to capture the knowledge of the experts. This knowledge has been gathered from secondary data. Although the principal methodology in medical secondary research is systematic review, commonly using meta-analytic statistical techniques, there are three general categories of research objectives: fact-finding, model building and database marketing [24] and from these, in our approach, we have chosen to use secondary data for model building, which involves specifying the relationships between two or more variables.

We examined the many surveys which have been conducted to investigate the relation between previously mentioned diabetic risk factors and complications to find relevant studies and collate their results. For that, available related knowledge was gathered from peer reviewed publications on clinical trials, meta-analysis and the Cochrane review from Medline, Cinahl, federated database searches and diabetes management guidelines published from 1981 to 2012. We also systematically searched the reference lists of included studies and of relevant reviews for potential studies. The results of surveys provided an inventory of knowledge on which to base our models. Three independent reviewers examined all titles of the resulting articles and rated each paper as ‘potentially relevant’, ‘of doubtful relevance’ or ‘not relevant’. Then the process was repeated for the first two groups by reviewing the abstracts and then the full-text versions and all ‘not relevant’ studies were omitted from the research. The chosen surveys were reviewed and the final results of every survey were taken out. The results from this review work were coded in Nvivo8 (a qualitative data analysis computer software package produced by QSR International) to manage and analyse associations and themes among diabetes predisposing factors and complications.

#### Data conversion

When secondary data are reported in a format that does not exactly meet the researcher’s needs, data conversion is necessary. All the various kinds of expressed data had to be converted into clear and concise records, with every record representing a relationship between one specific risk factor and one complication. Records had to be created in a format capable of being drawn on a scatter graph in order for us to be able to fit a curve on the scattered points. Therefore, data expressions had to be paraphrased into a table format as shown in Table 1.

**Table 1:** Format of the records to show the relationship.

Factor		Complication	
From (value)	To (value)	From (risk percentage)	To (risk percentage)

The reason for the choice of the ‘from-to’ format was to take into consideration the fact that the research has independently studied the relation between factors and complications. In other words, these tables are designed in a three-dimensional format in order to make data as clear as possible, but later on could be used as two-dimensional records. This format shows by how much the probability of a specific complication will change, if, independent of all other factors, the value of a specific risk factor changes.

Some studies express their observations exactly in our desired format. For instance, according to the DCCT research group [25], ‘A reduction in HbA1c from 11 to 9.9% yields a reduction in risk of retinopathy progression from 10.78 to 4.21 cases per 100 patient-years. In contrast, a reduction from 8 to 7.2% yields a reduction in risk from 2.43 to 1.48 cases per 100 patient-years’. Most of the studies, however, express their results in other formats. Some report only the change in severity occurring in a complication, but the absolute probability of that is not known. In such cases, by having the risk percentage of the first point, the risk percentage of other points could be calculated easily as the factors of change are available. A number of these studies demonstrate the changes in graphical representations, but the data from these needed to be extracted and presented in the format we needed.

### Applying prediction methods to make 1–1 relation models

The ultimate purpose of this step is to predict the probability of complications. Artificial neural networks (ANN) and least square regression analysis are two common techniques for making a prediction model based on observed data [26, 27]. These are the tools which have been used to create the models in this study.

#### Artificial Neural Networks (ANNs)

ANNs can be trained to predict numerical values such as probability, expected values, etc. This allows the systems to learn from past experience (examples) to recognize patterns in the gathered data. The system becomes more efficient with known results for large amounts of data [28]. Among the various neural network models, back propagation is the best general-purpose model and probably the best at generalization [29]. It is a supervised learning scheme by which a layered feed-forward network is trained to become a pattern-matching engine. The ANN takes a dataset and tries to combine the 'inputs' (factor value) in such a way as to model the 'output' (percentage of risk of the complication). These models can then be used on new data to predict what the output is likely to be for a given set of inputs. In this study we use the software application Tiberius [30].

#### Least square regression analysis

By far, the most widely used approach for numeric prediction is regression. In fact, many texts use the terms ‘regression’ and ‘prediction’ synonymously [31]. The objective of regression analysis is to determine the model that can best relate the output variable to various input variables. To produce the model using regression technique, a pattern which is likely to fit the data must be chosen first. There is a variety of patterns that might fit the observed data appropriately. Seven different patterns, i.e. linear, logarithmic, quadratic, cubic, power, s and exponential, have been chosen as the best options. SPSS 20 [32] is the software used in this study.

### Applying naïve Bayes to make n-1 relation models

Applying above mentioned methods, we would have a series of predictive models that indicate the relationship between each factor/complication pair. As we know, however, each complication is affected by a variety of factors. In order to see the effect of all risk factors on a single complication, we need to integrate all models created for that specific complication. For example, retinopathy is affected by HbA1c, albumin excretion rate and the duration of the diabetes. For every one of these factors a 1–1 model is created and then the models are integrated to make a 3–1 model which shows the effect of all the factors together on the risk of retinopathy. Because of the three dimensional data set tables (‘from-to’ format), every one of these models estimates the probability of retinopathy separately and independently of other factors.

In order to create n-1 models, we use the classification method. For each model, two classes are defined. Class one is to have that complication and the other class is lacking of that complication. The proposed model should be able to classify a new patient into one of these groups by observing his/her risk factors. Each n-1 model observes *n* factors of a patient and estimates how probable it is that s/he would be assigned to either of the two groups. In this study, naïve Bayes is selected as a classifier to create the models because it can deal with the independent factors.

#### Naïve Bayes

A naïve Bayesian classifier is a simple Bayesian classifier with strong independence assumptions. In simple terms, naïve Bayes assumes that the effect of an attribute value on a given class is independent of the values of the other attributes (class conditional independence).

Let ***X*** be a patient’s data set, which is described by a set of *n* attributes *f* (*f*_*1*,_ *f*_*2*,_…, *f*_*n*_). Let *H* be a hypothesis such as ‘patient ***X*** belongs to complication class *C’*. For classification, we want to determine *P*(*H*|***X***). Bayes’ theorem is useful in that it provides a way of calculating the posterior probability,*P*(*H*|***X***), from *P*(*H*), *P*(***X***|*H*) and *P*(***X***).

Bayes’ theorem is

$$P(H|X)=\frac{P\left(X|H\right)\;P\left(H\right)}{P\left(X\right)}$$

Bayes’ theorem is used in the naïve Bayesian classifier in the following way. Suppose that there are *m* classes of complication *C* (*C*1, *C*2,…, *Cm*). Given a patient’s data set, ***X***, the classifier will predict that ***X*** belongs to the complication class having the highest posterior probability, conditioned on ***X***. That is, the naïve Bayesian classifier predicts that patient ***X*** belongs to class *Ci* if and only if

$$P\left(C_{i}|X\right)>P\left(C_{j}|X\right)\;\text{for}\;1\leq j\leq \;m,j\neq i$$

Thus we maximize *P*(*C*_*i*_|**X**) [31]. By Bayes’ theorem

$$P\left(C_{i}\text{|}X\right)=\frac{\;P\left(X\text{|}C_{i}\right)\;P\left(C_{i}\right)}{P\left(X\right)}$$

Since *P*(***X***) is constant for all classes, only *P*(***X***|*C*_*i*_) *P*(*C*_*i*_) needs to be maximized. We compute the prior probabilities of the class *P*(*C*_*i*_) as

$$P\left(C_{i}\right)=\frac{\mathrm{Number of patients in the class}}{\mathrm{Total number of patients}}$$

Because the computation of *P*(***X***|*C*_*i*_) is extremely complex, especially for large data sets, the naive assumption of class conditional independence between attributes *f* is made. This presumes that the values of attributes *f* are conditionally independent of one another, given the complication class label of the patient. So,

$$P\left(X|C_{i}\right)=\overset{n}{\underset{k=1}{\prod }}P\left(f_{k}|C_{i}\right)=P\left(f_{1}|C_{i}\right)\times P\left(f_{2}|C_{i}\right)\times \ldots \times P\left(f_{n}|C_{i}\right)$$

As illustrated in the above equation, to calculate *P*(***X***|*C*_*i*_), the values of *P*(*f*_*k*_|*C*_*i*_) are needed. The 1–1 models created in the previous phase equals *P*(*C*_*i*_|*f*_*k*_) as they estimate the probability of a complication class (*C*_*i*_) on condition of observing a specific amount of a particular risk factor (*f*_*k*_). To calculate the value of *P*(*f*_*k*_|*C*_*i*_) from *P*(*C*_*i*_|*f*_*k*_) using the Bayes' theorem, we have

$$P\left(f_{k}|C_{i}\right)=\frac{P\left(f_{k}\right)}{P\left(C_{i}\right)}\times P\left(C_{i}|f_{k}\right)$$

Classes priors, *P*(*f*_*k*_) *and P*(*C*_*i*_) can be approximated with relative frequencies from the training data set:

$$\begin{array}{c} P\left(f_{k}\right)=\frac{\mathrm{Number of patients with specific value of the factor}\;f_{k}}{\mathrm{Total number of patients}}\\ P\left(C_{i}\right)=\frac{\mathrm{Number of patients in the class}}{\mathrm{Total number of patients}} \end{array}$$

In calculating the probabilities of *P*(*f*_*k*_) and *P*(*C*_*i*_) the patients should be counted only from those studies which are used to make the 1–1 models, but since we are using secondary data and there is no access to all original data-bases, we are obliged to use the data-base of the largest Australian longitudinal population-based study, AusDiab [23], instead.

The final product of this phase is determining the maximized class *C*_*i*_, to which patient ***X*** belongs on condition of observing attributes *f*_*1*_ to *f*_*n*_. This is an n-1 model as it points out how *n* factors affect a single complication.

Table 2 illustrates the observed information of one patient as an example to make an n-1 model.

**Table 2:** An example of observed information from a patient.

HbA1c	AER	Duration of disease	Retinopathy risk
7.8	21	8	?

*p*(*Ret*|*HbA1c*, *AER*, *Duration*) shows the 3–1 model to be created. Using the observations, we have to estimate the following probability:

$$\begin{array}{l} p(Ret=yes|HbA1c=7.8,\;AER=21,\;Duration=8)\\ \;=p\left(ret=yes\right)\times p\left(HbA1c=7.8|Ret=yes\right)\times p\left(AER=21|Ret=yes\right)\\ \;\times p\left(Duration=8|Ret=yes\right) \end{array}$$

For each factor we calculate the probability of *p*(*F*_*i*_|*Ret* = *yes*). For instance for HbA1c we have:

$$p(HbA1c=7.8|Ret=yes)=\frac{p\left(HbA1c=7.8\right)}{p\left(Ret=yes\right)}\;p(Ret=yes|HbA1c=7.8)$$

*p*(*Ret* = *yes*|*HbA*1*c* = 7.8) is calculated by using the 1–1 model created in the previous phase. *p*(*Ret* = *yes*) and *p*(*HbA*1*c* = 7.8) are calculated simply by using AusDiab data, as mentioned in Section 3.3.1. For that we need to calculate *p*(*Ret* = *yes*) and *p*(*HbA*1*c* ∈ ]7, 8]) in all diabetic patients. We performed this calculation for all risk factors and complications which are selected in this study. Then, *p*(*Ret* = *yes*|*HbA*1*c* = 7.8, *AER* = 21, *Duration* = 8) can be calculated once all probabilities are available.

### Applying a Bayesian network to make an n-k relation network

Unlike naïve Bayesian classification (which assumes class conditional independence), Bayesian belief networks allow class conditional independencies to be defined between subsets of variables. Bayesian networks [33] are particularly good at providing a powerful and conceptually transparent formalism for probabilistic modelling and they are typically well suited for representing medical knowledge.

A Bayesian network is a directed acyclic graph (DAG) on which a probability distribution is overlaid. The nodes of the graph represent random variables or events. Each variable consists of a finite set of mutually exclusive states. It is possible for variables to have a continuous state, representing a numerical value, but there are several limits on their use, so we convert continuous variables into discrete ones. The directed links between variables in the graph represent causal relationships.

Each variable has a conditional probability table (CPT) associated with it. Variables with no parents (risk factors) do not have any probability table as they are observed in the patient. Variables with parents (complications) have conditional probability tables, which give a probability distribution for every combination of states of the variable’s parents.

There is a wide variety of Bayesian network software programs available [34]. One of the most popular commercial programs in this area is Netica [35], from Norsys Software Corp, which is used in this study. It provides a simple graphical user interface that can be used for both creating and running a network.

Building a Bayesian network involves three major steps. First, the set of relevant variables and their possible values must be decided. Next, the network structure must be built by connecting the variables into a DAG. Finally, the CPT for each network variable must be defined.

#### Constructing the graphical model

To construct a Bayesian network, the hypothesis variables should be determined first. These are variables for which the probability distribution is to be calculated. In this study the hypothesis variables are complications. Next, evidence variables are added. These variables represent the factors that will be observed in patients. Once observed, these variables allow the information to be entered into the network. The network is formed by linking these variables using directed edges (arrows).

#### Constructing the probability tables

Each complication has a table which shows its probability for various degrees of different risk factors. The tables are filled by using the 1–1 and n-1 models created in previous phases. To deal with continuous values, we convert them into discrete variables by defining a range for each group. The probability of the complication will be calculated for all possible states of risk factors and will be entered into the table. Table 3 is an example which shows how the probability of retinopathy is determined by different values of HbA1c, blood pressure, diabetes duration, BMI and smoking.

**Table 3:** Format of the probability table for retinopathy.

HbA1c	Blood pressure	Diabetes duration	BMI	Smoking	Retinopathy Risk
8–9	110–120	10–15	15–18.4	Sometimes	54%
7–8	130–140	0–5	22.9–27.5	No	46%

### Model validation

Validation is an important stage in a system’s development. Although several evaluation theories have been developed for application in different fields of expert systems in medicine, there is no consensus on the best way to evaluate advice from expert systems in diabetes [36].

#### Evaluation of gathered data

One of the disadvantages of secondary data is that the user has no control over their accuracy. Research conducted by others may be biased to support the vested interest of the source. If the possibility of bias exists, the secondary data should not be used. If the accuracy of the data cannot be established, the researcher must determine whether using the data is worth the risk.

We accepted data from reliable studies such as the Diabetes Control and Complications Trial and follow-up Study (DCCT) and the United Kingdom Prospective Diabetes Study (UKPDS). Nevertheless, we assessed the reputation of the journals or conferences which published the data and finally we did cross-checks of data, that is, the comparison of data from one source with data from another source to determine the similarity of independent projects.

#### 1–1 model comparison and testing

The accuracy of a predictor refers to how well a given predictor can guess the value of the predicted attribute for new or previously unseen data.

It is important that we assess how well the model fits the actual data. We do this because even though this model is the best one available, it can still be a bad fit to the data. This is easily calculated by *R* ^2^:

$$R^{2}=\frac{SS_{M}\;\left(\text{model sum of squares}\right)}{SS_{T}\;\left(\text{total sum of squares}\right)}$$

A second way of assessing the model is through the *F*-test. *F* is based upon the ratio of the improvement due to the model (SS_M_) and the difference between the model and the observed data (SS_R_). Actually, because the sums of squares depend on the number of differences that we have added up, we use the average sums of squares (‘mean squares’ or MS). To work out the mean sums of squares, we divide by the degrees of freedom. For SS_M_, the degrees of freedom are simply the number of variables in the model, and for SS_R_ (residual sum of squares) they are the number of observations minus the number of parameters being estimated. The result is the mean squares for the model (MS_M_) and the residual mean squares (MS_R_).

$$F=\frac{MS_{M}}{MS_{R}}$$

It is important to know that the *F*-ratio tells us how much variability the model can explain relative to how much it cannot explain. A good model should have a large *F*-ratio (greater than 1 at least).

As a second part of this step, we have to assess how well our model can predict the outcome in a different sample. To test whether the model can generalize, we can look at cross-validating it. Assessing the accuracy of a model across different samples is known as cross-validation. In this research, not only are the values of *R* ^2^ calculated, but also an adjusted *R* ^2^. This adjusted value indicates the loss of predictive power. Whereas *R* ^2^ tells us how much of the variance in *Y* is accounted for by the predictive model from our sample, the adjusted value tells us how much variance in *Y* would be accounted for if the model had been derived from the population from which the sample was taken. SPSS derives the adjusted *R* ^2^ using Wherry’s equation [37]. This equation, however, has been criticized because it tells us nothing about how well the regression model would predict an entirely different set of data. One version of adjusted *R* ^2^ that does tell us how well the model cross-validates uses Stein’s formula [38] which is:

$$adjusted\;R^{2}=1-\left[\left(\frac{n-1}{n-k-1}\right)\left(\frac{n-2}{n-k-2}\right)\left(\frac{n+1}{n}\right)\right]\;\left(1-R^{2}\right)$$

In Stein’s equation, *R* ^2^ is the unadjusted value, *n* is the number of participants and *k* is the number of predictors in the model.

In addition, there is an independent errors assumption in regression, which means that for any two observations the residual terms should be uncorrelated (or independent). This eventuality is sometimes described as a lack of ‘autocorrelation’. This assumption can be tested with the Durbin–Watson test, which tests for serial correlations between errors. The test statistic can vary between 0 and 4, with a value of 2 meaning that the residuals are uncorrelated. As a general rule, results between 1.5 and 2.5 imply independent errors [39].

#### n-1 and n-k model validation

Suppose that one has a data set including 10 positive and 90 negative samples. A simple decision model which classifies all the instances as negative would represent 90% accuracy, whereas it could not correctly predict any positive instance. From a medical point of view, a misclassified negative is the most critical decision, because the patient would not have appropriate medical care in that case. One also needs to reduce the number of misclassified positives, however, which leads to unnecessary additional physical examination or treatment. For classifiers, sensitivity, specificity and positive predictive value are useful alternatives to the accuracy measure.

Sensitivity is the proportion of positive samples that are correctly identified, while specificity is the proportion of negative samples that are correctly identified.

$$\begin{array}{c} \text{Sensitivity}=\frac{\sum \;\text{True Positive}}{\sum \;\text{All Positive}}\\ \text{Specificity}=\frac{\sum \;\text{True Negative}}{\sum \;\text{All Negative}} \end{array}$$

In addition, we may use positive predictive value or precision to access the percentage of samples labelled as a ‘class’ that actually are in that ‘class label’ group.

$$\text{Positive predictive value}=\frac{\sum \;\text{True Positive}}{\sum \;\text{Model outcome Positive}}$$

We use the real data of diabetic patients from the AusDiab research study to validate our final model. For that, we define several cut-off points to convert the continuous probability of each diabetes complication into two discrete groups (yes or no) and then calculate all mentioned measures for the model.

## Results

### Gathered knowledge

NVivo8 is used to organize and analyse associations and themes related to predisposing factors and chronic complications. Detailed descriptions of that have been included in a previous report [40]. In brief, both chronic diabetes complications and diabetes predisposing factors have been classified hierarchically by 27 and 47 nodes, respectively, based on existing endocrinology text books [41–43]. The two are connected by 126 relationships, categorized into three different types: ‘is a cause for’, ‘prevents from or decreases’ and ‘are the same’. All these nodes and relationships are supported by more than 590 identified facts from 99 different sources including 47 prospective or retrospective longitudinal studies (containing more than 450,000 patient/years observations) and also 21 cross-sectional studies (containing more than 22,500 cases). (Details are available in the supplementary files)

### Codified knowledge

Next, data tables were created based on the format explained in Table 1. As mentioned, the reason we chose the ‘from-to’ format was to take into consideration the fact that the research has independently studied the relation between factors and complications, but here we use them as two-dimensional records. Table 4, as an example, shows a dataset of the relation between HbA1c and non-proliferative diabetic retinopathy (NPDR).

**Table 4:** A dataset showing the relation between HbA1c and NPDR.

HbA1c level	Risk of NPDR
6.8	14.5
6.95	3
7.85	20
7.95	3.8
8	14
8.95	7.1
9.2	27
9.5	20
9.9	27
9.95	7.9
10.5	9.9
10.55	28
11.7	51
12	32
13	32
13.7	40

Mean arterial pressure (MAP) is used to represent the blood pressure level, applying this equation:

$$MAP≅DP+\frac{1}{3}\left(SP-DP\right)$$

where SP and DP are systolic and diastolic pressures, respectively.

### 1–1 Relation Models

In order to create the 1–1 models, all 15 data tables are used to perform curve fitting. We develop models using two different software applications, Tiberius [30] and SPSS [32], which work based on ANN and least square regression, respectively.

#### Artificial neural networks (ANNs)

**Fig 2:**
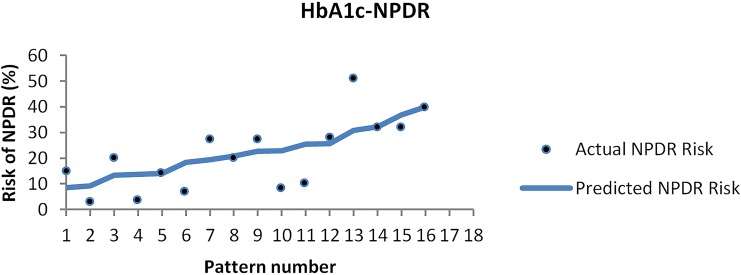
The models made by the neural network (HbA1c-all).

The ANN model produced by the software based on dataset Table 4 (between HbA1c and NPDR) is presented in Fig 2 as an example.

Table 5 shows the statistical indices of ANN patterns for all dataset tables.

**Table 5:** The statistical indices of ANN patterns.

Related data table	Std. Error of the Estimate	R	R Square
HbA1c-PDR	9.288	0.748	0.560
HbA1c-NPDR	9.016	0.735	0.540
HbA1c-DR	13.317	0.739	0.546
HbA1c-Micro	8.147	0.796	0.633
HbA1c-Macro	4.391	0.693	0.480
Dur-PDR	10.522	0.792	0.628
Dur-NPDR	9.509	0.898	0.807
Dur-DR	16.254	0.854	0.729
Dur-Micro	8.516	0.411	0.169
Dur-Macro	2.730	0.961	0.924
BP-Micro	9.850	0.523	0.273
BP-Macro	3.977	0.681	0.464
AER-PDR	6.515	0.931	0.866
AER-NPDR	8.657	0.741	0.549
AER-DR	9.819	0.865	0.748

#### Regression analysis

**Fig 3:**
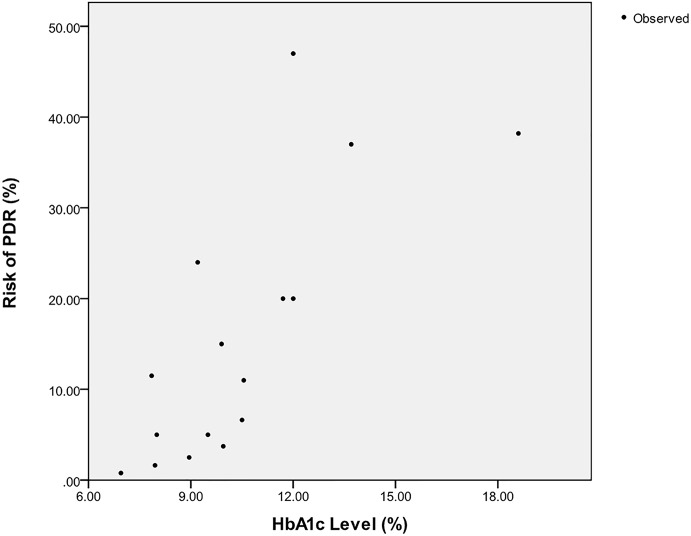
A scatter graph between HbA1c level and risk of PDR.

Seven different types of regression model, i.e. linear, logarithmic, quadratic, cubic, power, and exponential, have been chosen to build the data fitting model for scatter graph illustrated in Fig 3. We entered all dataset tables in SPSS.

**Fig 4:**
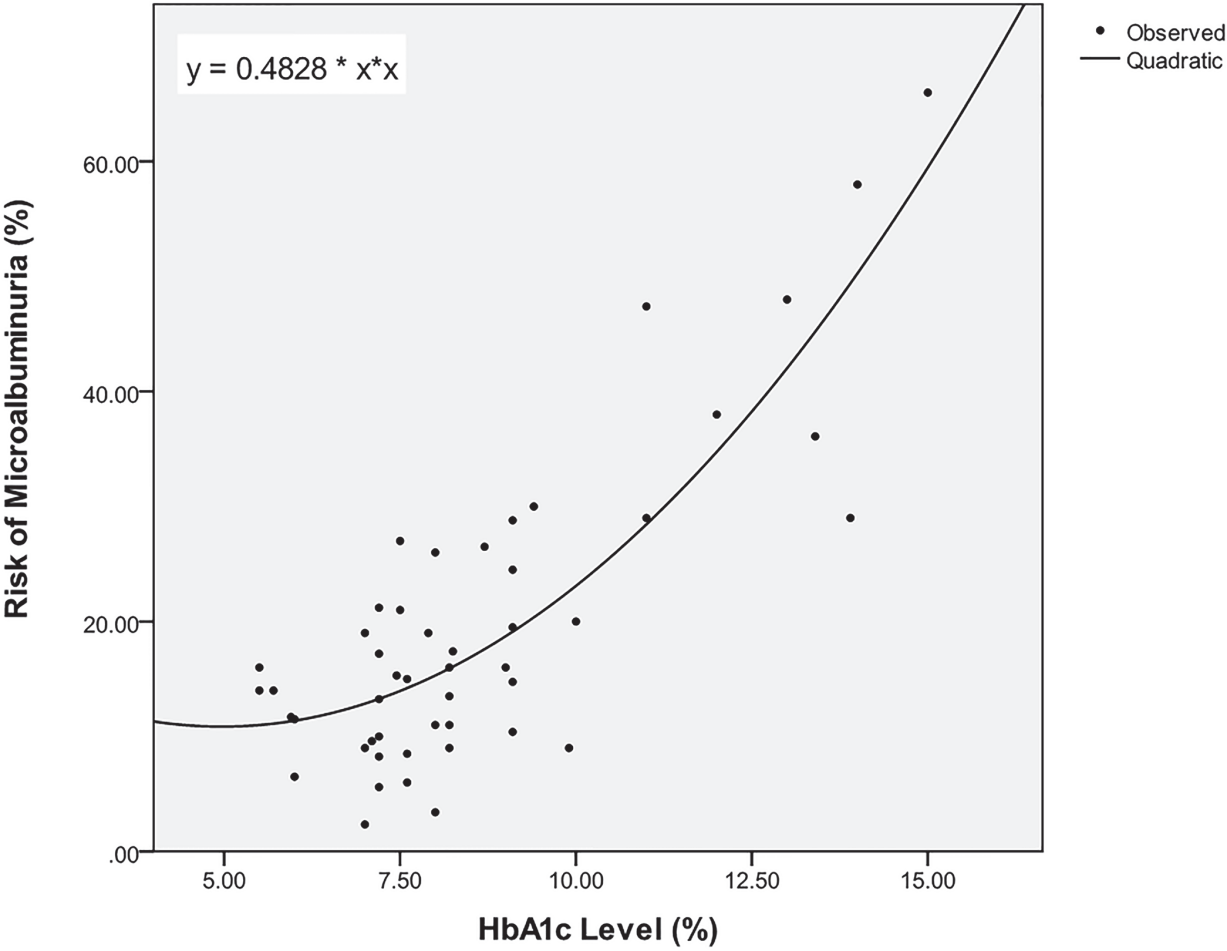
The best fitted function (quadratic) to the HbA1c-Micro set of data.

Fig 4 shows the scatter graph along with plots of the selected best fitted function to the set of data values among all seven patterns for the HbA1c-Micro set of data.

SPSS calculates the values of parameters for all mentioned patterns to achieve the functions. Table 6 is an example showing the statistical indices of these seven patterns for HbA1c-Micro dataset tables. The highlighted row in the table indicates the selected best fitted function to the set of data values among all seven patterns.

**Table 6:** Statistical indices of selected seven patterns for HbA1c-Micro data table.

Models	R	R Square	Wherry’s Adjusted R Square	Std. Error of the Estimate	F-ratio	Sig
Linear	0.804	0.647	0.639	8.166	85.961	< 0.001
Logarithmic	0.761	0.579	0.570	8.911	64.662	< 0.001
Quadratic	0.834	0.695	0.681	7.671	52.350	< 0.001
Cubic	0.835	0.697	0.676	7.732	34.444	< 0.001
Power	0.642	0.412	0.400	0.527	32.946	< 0.001
S	0.597	0.357	0.343	0.551	26.045	< 0.001
Exponential	0.666	0.444	0.432	0.512	37.560	< 0.001

### n-1 relation models

By using the naïve Bayes theorem we can calculate the probability of a complication by observing the risk factors of a patient.

### n-k relation models

In this phase the final model is developed as a network which shows how risk factors and complications affect each other. Firstly, the Bayesian network should be constructed.

#### Graphical model

**Fig 5:**
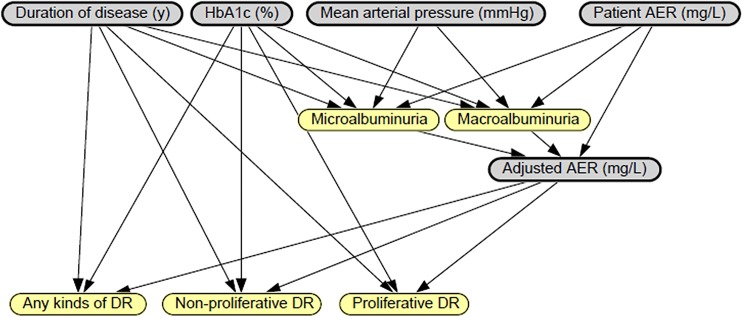
Bayesian network created by factors and complications.

First, the factors and complications are drawn in circles. Then, we use the relationships created by Nvivo8 to link the variables by directed edges. As illustrated in Fig 5, all complications are directed by all factors. Retinopathy is affected by microalbuminuria and macroalbuminuria, as two complications.

#### Constructing the probability tables

**Fig 6:**
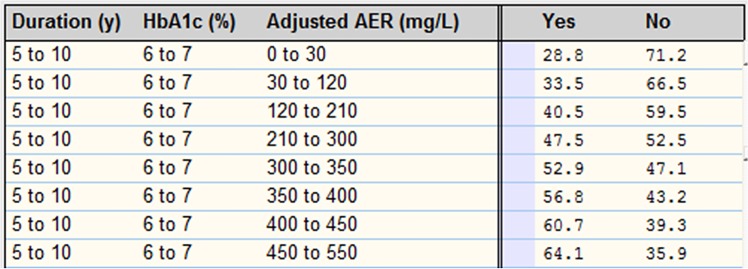
A piece of probability table for DR.

**Fig 7:**
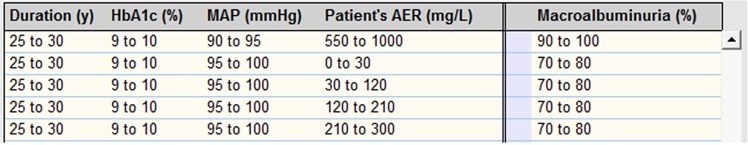
A piece of probability table for macroalbuminuria.

As mentioned before, each complication has a table which shows its probability for various amounts of parental risk factors. This table consists of rows that are combinations of all possible states of all risk factors. So, the number of rows is equal to multiplying the number of states of all factors together. For each row, the probability of complication is calculated by using an n-1 model as explained in the previous phase. Fig 6 and Fig 7 illustrate a piece of the probability table of the complications DR and macroalbuminuria, respectively.

#### Using the network

**Fig 8:**
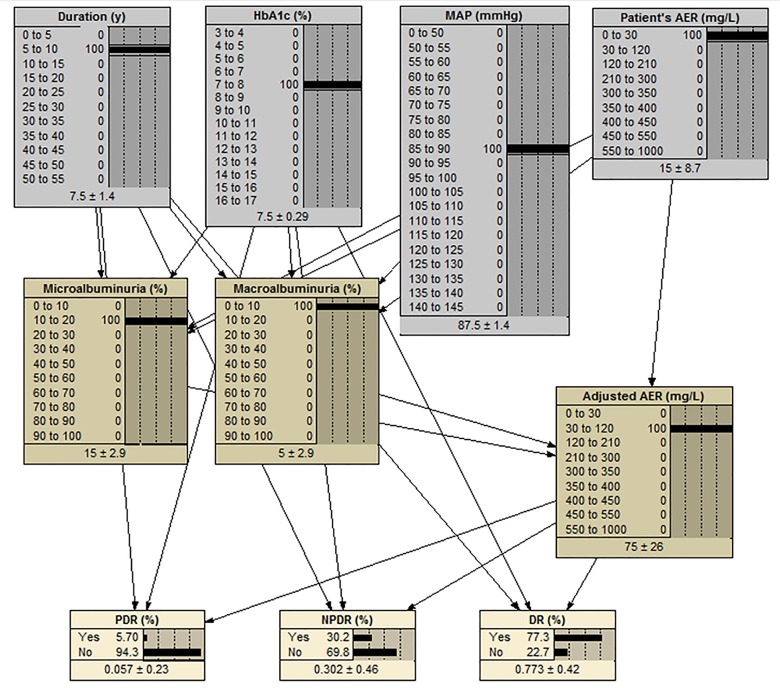
A Bayesian network calculates the probability of complications for a patient.

Once the probability table of each complication is created, the network is ready to be compiled. The compiled network is able to get risk factors as input (observation) and estimate the probability of all complications. This network is bringing n risk factors and k complications together, so it is called the n-k model. Fig 8 shows the compiled network calculating the probability of complications for the patient in Table 6.

### Model evaluation

#### Model comparison and testing

We applied the Durbin-Watson test for all six dataset tables and all the results were between 1.5 and 2.5, which implies the existence of independent errors [39]. Then, using R-squared, it can be clearly seen that the best selected regression models fit the data in all 15 dataset tables better than ANN, as well as the other six regression patterns (Table 7).

**Table 7:** Statistical details of ANN and the best fitted regression models.

	ANN	Best fitted regression patterns						
Dataset table	R^2^	R^2^	Durbin-Watson	Wherry’sAdj-R2	Stein’s Adj-R2	F ratio	Sig.	Patternshape
HbA1c-PDR	.560	.628	1.923	.602	.544	23.7	<. 001	S
HbA1c-NPDR	.540	.542	2.104	.509	.439	16.6	.001	linear
HbA1c-DR	.546	.589	2.253	.580	.561	64.5	<. 001	linear
HbA1c-Micro	.633	.695	1.520	.681	.668	52.4	<. 001	quadratic
HbA1c-Macro	.480	.801	1.964	.612	.547	21.6	.001	S
Dur-PDR	.628	.701	1.822	.685	.668	43.3	<. 001	quadratic
Dur-NPDR	.807	.868	2.396	.857	.833	79.2	<. 001	power
Dur-DR	.729	.731	1.545	.725	.713	122.3	<. 001	linear
Dur-Micro	.169	.374	1.580	.360	.333	27.5	<. 001	logarithmic
Dur-Macro	.924	.926	2.055	.919	.902	125.0	<. 001	linear
BP-Micro	.273	.456	1.803	.427	.367	15.9	.001	exponential
BP-Macro	.464	.631	2.380	.600	.533	20.5	.001	linear
AER-PDR	.866	.926	1.770	.906	.869	46.1	<. 001	cubic
AER-NPDR	.549	.585	1.801	.544	.451	14.1	.004	linear
AER-DR	.748	.766	2.136	.743	.690	32.8	<. 001	linear

### n-1 and n-k model validation

Validation is a measurement of correctness in a real environment or by means of real data. In order to validate the final model, we randomly selected several samples of diabetic patients’ data from the AusDiab research study. For that, 84 random cases have been selected and their model estimated risk of complications was compared with their real outcomes and then computed the sensitivity, specificity and precision rate of model for all five complications. Five different cut-off points (60%, 70%, 80%, 90% and 100%) have also been considered, in order to translate the percentage of complication probability into their presence or absence. The averages for sensitivity, specificity and precision were 80%, 85% and 60%, respectively. Except for NPDR, the best cut-off point for all complications was 100%, while it was 60% for NPDR risk prediction (Table 8).

**Table 8:** Sensitivity, specificity and precision rate of the model for all five complications.

	Cut off %	Sensitivity	Specificity	Precision
Micro(84 cases)	60	87.2	97.8	97.1
	70	87.2	100	100
	80	87.2	100	100
	90	87.2	100	100
	100	87.2	100	100
Macro(84 cases)	60	70	91.9	53.9
	70	70	91.9	53.9
	80	70	91.9	53.9
	90	70	94.6	63.7
	100	70	94.6	63.7
DR(84 cases)	60	93.3	59.3	56
	70	90	61.1	56.3
	80	83.3	66.7	58.1
	90	83.3	68.5	59.5
	100	80	70.4	60
NPDR(28 cases)	60	70	77.8	63.6
	70	60	77.8	60
	80	60	77.8	60
	90	50	77.8	55.6
	100	40	77.8	50
PDR(28 cases)	60	100	84	42.9
	70	100	84	42.9
	80	100	84	42.9
	90	100	84	42.9
	100	100	88	50

## Conclusion and Discussion

Early and appropriate intervention in diabetes is able to reduce the rate of complications, to prolong life expectancy and to reduce the financial cost [44]. The most common predictive risk assessment models for diabetes complications, however, are not able to deal with all the major complications, but are mainly focused on cardiovascular diseases, coronary heart disease and diabetic retinopathy [45]. In most of these studies only relatively simple statistical approaches, such as additive scores or logistic regression assuming independence between variables, have been applied [46].

In this research, we gathered the observed data from previously conducted surveys which investigate the relation between HbA1c, duration of disease, AER and blood pressure level, and diabetic micro- and macroalbuminuria and any kinds of retinopathy using NVivo8 to organize data and this enabled us to use and follow up the data from more than 450,000 patient/years. Then we made the data uniform and constructed a table structure for them. In addition, because of its modular structure, the final model is flexible enough to include all chronic complications which have been previously studied including microvascular complications (retinopathy, neuropathy and nephropathy) as well as macrovascular complications (coronary heart diseases, cerebrovascular diseases and peripheral vascular diseases).

Curve fitting was performed using two different software applications, Tiberius [30] and SPSS [32]. ANN created one model and regression analysis produced seven models using seven different patterns, i.e. linear, logarithmic, quadratic, cubic, power, s and exponential. These have been chosen as the best options. To see how well the 1–1 models fit the observed data, we assessed them using R^2^ and F-test. R squared was between 0.374 and 0.926, and the F-ratio was between 14 and 125 (greater than one) and significant at *p* ≤ 0.001 for all models, thus indicating statistically acceptable results. To test whether the 1–1 models generalize, we used cross-validation and calculated adjusted R^2^. R^2^ comparison suggests that the best fitted regression techniques provide more accurate models than ANN or the other six regression patterns. As ANN becomes more efficient with known results for large amounts of data, it is not surprising that regression patterns outperform ANN in all models because of the small dataset tables we have. We also applied the Durbin-Watson test for all the dataset tables and all the results were between 1.5 and 2.5. This implies the existence of independent errors.

In order to determine the probability of each complication, all its related models were integrated to make an n-1 model using the naïve Bayes theorem. A random set of real patient data from AusDiab research has been used to assess the validity of the final model. The range of sensitivity and specificity was between 70 and 100 percent. This was between 53 and 100 percent for positive predictive value, thus indicating a very high level of success in the prediction of albuminuria.

### Significant findings of the research

This model has benefits for diabetic patients and the health workers who are involved in diabetes diagnosis and treatment. It can be used to inform diabetic patients about the risk and severity of probable complications, to help health advisors to convince patients to change their lifestyle, and to inform healthcare providers so they can design immediate preventive interventions before a patient loses her/his capability. The contributions of this research are:

- It proposes a divide-and-conquer technique to overcome the complexities of a very intricate relationship.
- It proposes a method to gather and standardize the knowledge from other research and devise a structured format for it.
- It creates a predictive model to indicate the relationship between individual risk factors and complications.
- It suggests a method to integrate a number of different models and derive probability tables from them.
- It proposes the application of a Bayesian network to relate all of the factors to diabetic complications.
- This model which is, then, developed, was tested by real data and was shown to be statistically acceptable.

### Limitations

In data conversion, different measurement units, different statistical formats of data calculation or presentation and different study conditions (such as the number of cases, duration of follow-up, etc.) were some of the obstacles we needed to overcome. Because of the time-consuming process of contacting the authors, we omitted the study wherever data conversion was not possible using the published result.

Another limitation of this study was difficulties in accessing the raw data of the patients that is necessary to weigh the finding of these studies. So, this research considers all the findings with the same degree of importance.

In making the n-1 models, we need to calculate some probabilities from the same data-bases which are used to make the 1–1 models. But since we are using secondary data and access was not available to all original data-bases, we were obliged to use the database of the AusDiab, instead.

The next limitation is that when we use the regression models for data beyond the training dataset extrema, we cannot be sure about the accuracy of the model when we are working on the data in these gaps.

## Future Work

We extracted a full list of risk factors and complications (37 risk factors and 19 complications) [40], but hasn’t map out all the relationship (fifteen 1–1 modelling has been done). From here, the first step is to gather adequate data to demonstrate the relation between all the risk factors and complications through an extensive literature review. Then, 1–1, n-1 and n-k modelling processes need to be performed to create the completed final model. Next, more experimental research with a real time data must be used in order to improve accuracy. One way of doing this would be to integrate the model with a regional electronic health database and design a longitudinal survey considering both patients’ real data and health workers’ feedback.

In future, this methodology could also be applied to other multi-factorial chronic diseases. Extracting a complete list of predisposing factors and complications, data gathering about the relationships between these two groups, data conversion, 1–1 modelling, n-1 modelling and n-k modelling could be applicable for all such diseases. Given enough related data, it may even be possible to design a model for primary prevention of diabetes.

## Supporting Information

S1 File General Introduction and Review of Decision Support Systems used for Diabetes. (DOCX)

S2 File A list of refrences used to make dataset tables. (DOCX)

S3 File ACCESS TO DATA, BIOLOGICAL MATERIALS AND SUBJECTS. (DOCX)
