# Supplementary material for: Applying a Novel Combination of Techniques to Develop a Predictive Model for Diabetes Complications
Source: PLoS One. 2015 Apr 22;10(4):e0121569. doi: 10.1371/journal.pone.0121569 (PMC4406519; doi:10.1371/journal.pone.0121569)
Supplement: S1 File — (DOCX) [file pone.0121569.s001.docx]

**Supplementary File 1**

**General Introduction and Review of Decision Support Systems used for Diabetes:**

Among the rest, applying DSS is considered to be one of the best methods to improve the management of diabetes’ chronic complications.

A variety of decision support systems are available in the area of diabetes management [1,2,3,4,5,6,7], however, there are only a few examples of risk advisory tools in the domain of diabetes secondary prevention, which are not able to deal with all the major complications, but are mainly focused on cardiovascular diseases, coronary heart disease and diabetic retinopathy [8].

Skevofilakas et al. [9], for example, created a decision support system able to predict the risk of a Type 1 diabetic patient developing retinopathy. The UKPDS risk engine [10] calculates the complications of type 2 diabetes based on age, sex, ethnicity, smoking status, presence or absence of atrial fibrillation, levels of HbA1c, systolic blood pressure, total cholesterol and HDL cholesterol. It provides risk estimates for non-fatal and fatal coronary heart disease or stroke. Diabetes PHD [11] was an online tool that calculated the percentages of risk for diabetes and 5 complications associated with it. The model was populated with thousands of simulated people, all of whom were living simulated lives, sometimes developing simulated diabetes and/or its complications. The Diabetes PHD calculator gave percentages of risk for diabetes and 5 complications of diabetes. Cleveland [12] was a predictive model able to estimate the risk of death in Type 2 diabetic patients, employing a Cox regression. Finally, a Cox regression model [13] for estimating the absolute 5-year risk of first incident fatal and nonfatal CVD in Type 2 diabetic patients was derived from data in Sweden.

References

1. Lehmann E D, Deutsch T, Carson E R, Sonksen P H (1994) Aida: An interactive diabetes advisor. Computer Methods and Programs in Biomedicine 41: 183.

2. Narasinga Rao M R, Sridhar G R, Madhu K, Rao A A (2010) A clinical decision support system using multi-layer perceptron neural network to predict quality of life in diabetes. Diabetes and Metabolic Syndrome: Clinical Research and Reviews 4: 57.

3. Zahlmann G, Franczykova M, Henning G, Strube M, Huttl I, Hummel I, et al. (1990) Diabetex - a decision support system for therapy of type i diabetic patients. Computer Methods and Programs in Biomedicine 32: 297.

4. Awad E M, Tremaine M D (2003) Diabetic foot advisor: An empirical investigation using expert system.(author abstract). International Journal of Healthcare Technology and Management 1: 106.

5. Tudor R S, Hovorka R, Cavan D A, Meeking D, Hejlesen O K, Andreassen S (1998) Dias-niddm-a model-based decision support system for insulin dose adjustment in insulin-treated subjects with niddm. Computer Methods and Programs in Biomedicine 56: 175.

6. Hejlesen O K, Andreassen S, Hovorka R, Cavan D A (1997) Dias-the diabetes advisory system: An outline of the system and the evaluation results obtained so far. Computer Methods and Programs in Biomedicine 54: 49.

7. Montani S, Bellazzi R, Portinale L, Stefanelli M (2000) Evaluating a multi-modal reasoning system in diabetes care. Advances in case-based reasoning: 5th european workshop, ewcbr 2000 trento, italy, september 2000 proceedings. pp. 213.

8. Koumakis L, Chiarugi F, Lagani V, Kouroubali A, Tsamardinos I (2012) Risk assessment models for diabetes complications: A survey of available online tools. In: Nikita K, Lin J, Fotiadis D, Arredondo Waldmeyer M-T, editors. Wireless mobile communication and healthcare: Springer Berlin Heidelberg. pp. 46.

9. Skevofilakas M, Zarkogianni K, Karamanos B G, Nikita K S (2010) A hybrid decision support system for the risk assessment of retinopathy development as a long term complication of type 1 diabetes mellitus. Conf Proc IEEE Eng Med Biol Soc 2010: 6713.

10. Stevens R J, Kothari V, Adler A I, Stratton I M (2001) The ukpds risk engine: A model for the risk of coronary heart disease in type ii diabetes (ukpds 56). Clin Sci 101: 671.

11. Eddy D M, Schlessinger L (2003) Validation of the archimedes diabetes model. Diabetes Care 26: 3102.

12. Wells B J, Jain A, Arrigain S, Yu C, Rosenkrans W A, Kattan M W (2008) Predicting 6-year mortality risk in patients with type 2 diabetes. Diabetes Care 31: 2301.

13. Cederholm J, Eeg-Olofsson K, Eliasson B, Zethelius B, Nilsson P M, Gudbjornsdottir S (2008) Risk prediction of cardiovascular disease in type 2 diabetes: A risk equation from the swedish national diabetes register. Diabetes Care 31: 2038.
