# Supplementary material for: Applying a Novel Combination of Techniques to Develop a Predictive Model for Diabetes Complications
Source: PLoS One. 2015 Apr 22;10(4):e0121569. doi: 10.1371/journal.pone.0121569 (PMC4406519; doi:10.1371/journal.pone.0121569)
Supplement: S2 File — (DOCX) [file pone.0121569.s002.docx]

**Supplementary File 2**

A list of refrences used to make dataset tables

| Author / Name of study | Type of study | Number of cases | Duration | Country | Relation |
| --- | --- | --- | --- | --- | --- |
| Brownlee, Aiello et al. 2011 [1] | Textbook of Endocrinology | - | - | US | list of risk factors and complications |
| DCCT 1993 [2] | Randomised controlled trial | 1441 | 6.5 years | US | HbA1c/Retinopathy & Albuminuria |
| Klein, Klein et al. 1984 [3] | Cross Sectional | 996 | - | US | Duration/Retinopathy |
| DCCT 2000 [4] | Randomised controlled trial | 1208 and 1302 | 10.5 and 9.5 years | US | HbA1c & Duration/ Retinopathy & Albuminuria |
| DCCT 1995 [5] | Randomised controlled trial | 1441 | 6.5 years | US | HbA1c/Retinopathy |
| UKPDS 1998 [6] | Randomised controlled trial | 5102 | 20 years | UK | HbA1c/Retinopathy & Albuminuria |
| Chase, Jackson et al. 1989 [7] | Retrospective random trial | 230 | - | US | HbA1c & Duration/ Retinopathy & Albuminuria |
| Krolewski, Canessa et al. 1988 [8] | Retrospective random trial | 89 | 14 years | US | Blood pressure/ Albuminuria |
| Krolewski, Warram et al. 1986 [9] | Retrospective study | 292 | 20-40 years | US | Duration/Retinopathy |
| Klein, Moss et al. 1990 [10] | Prospective random trial | 129 | 4 years | US | Duration & Blood pressure/Retinopathy |
| Kostraba, Klein et al. 1991 [11] | Cross Sectional | 657 | - | US | Duration & Blood pressure/Retinopathy & Albuminuria |
| Klein, Moss et al. 1990 [12] | Prospective study | - | - | US | HbA1c/Retinopathy |
| Al-Futaisi, Al-Zakwani et al. 2006 [13] | Cross sectional | n=261 | - | Oman | HbA1c & Blood pressure & Albuminuria/Albuminuria |
| Thai Multicenter Research Group on Diabetes Mellitus 1994 [14] | Cross Sectional | 2060 | - | Thailand | Blood pressure/ Retinopathy |
| UKPDS 1998 [15] | Randomised controlled trial | 1148 | 8.4 years | UK | Blood pressure /Retinopathy & Albuminuria |
| Kullberg and Arnqvist 1993 [16] | Prospective study | 172 and 186 | 7.9 years | Sweden | HbA1c/Retinopathy & Albuminuria |
| Krolewski, Laffel et al. 1995 [17] | Cross Sectional | 1613 | - | US | HbA1c & Duration/ Albuminuria |
| Savage, Estacio et al. 1996 [18] | Prospective study | 947 | 5 years | US | Albuminuria/Retinopathy |
| Fujisawa, Ikegami et al. 1999 [19] | Cross Sectional | 294 | - | Japan | Blood pressure & Duration & Albuminuria/ Retinopathy |
| Mogensen and Christensen 1984 [20] | Prospective observational | 44 | 14 years | US | Albuminuria/Albuminuria |
| Bruce, Linda et al. 2003 [21] | Prospective observational | 386 | 8 years | US | Blood pressure & Duration & HbA1c/ Albuminuria |
| Hovind, Tarnow et al. 2004 [22] | Prospective observational | 286 | 18 years | Denmark | Blood pressure & Albuminuria & HbA1c/ Albuminuria |
| Mogensen 1982 [23] | Prospective study | 6 | 73 months | Denmark | Blood pressure/ Albuminuria |
| Dorsey 2006 [24] | Thesis | - | - | US | list of risk factors and complications |
| DCCT 1996 [25] | Randomised controlled trial | 1441 | 6.5 years | US | HbA1c/Retinopathy & Albuminuria |
| Gæde, Vedel et al. 2003 [26] | Randomised controlled trial | 160 | 7.8 years | Denmark | HbA1c & Blood pressure /Retinopathy & Albuminuria |
| Nathan, Cleary et al. 2005 [27] | Randomised controlled trial | 1394 | 17 years | US | HbA1c/Albuminuria |
| Olsen, SjÃ¸lie et al. 2000 [28] | Prospective study | 339 | 6 years | Denmark | Albuminuria & Duration & HbA1c/Retinopathy & Albuminuria |
| Romero-Aroca, Baget-Bernaldiz et al. 2011 [29] | Prospective study | 297 | 10 years | Spain | HbA1c & Blood pressure & Albuminuria /Retinopathy |
| Okudaira, Yokoyama et al. 2000 [30] | Retrospective study | 394 | 7.1 years | Japan | HbA1c & Blood pressure & Duration/Retinopathy |
| Golbahar, Rahimi et al. 2008 [31] | Cross Sectional | n=254 | - | Iran | HbA1c & Blood pressure & Duration/Retinopathy |
| Imano, Miyatsuka et al. 2001 [32] | Cross Sectional | 162 | - | Japan | Duration/Retinopathy |
| Tam, Lam et al. 2009 [33] | Prospective study | 354 | 4.2 years | China | HbA1c & Albuminuria /Retinopathy |
| Semeraro, Parrinello et al. 2011 [34] | Prospective study | 5034 | 11 years | Italy | HbA1c & Blood pressure & Duration & Albuminuria/Retinopathy |
| Massin, Erginay et al. 2007 [35] | Cross Sectional | 504 | - | France | HbA1c & Blood pressure & Duration/Retinopathy |
| Rema, Ponnaiya et al. 1996 [36] | Cross Sectional | 6792 | - | India | HbA1c & Blood pressure & Duration/Retinopathy |
| Romero, Salvat et al. 2007 [37] | Prospective study | 112 | 15 years | Spain | HbA1c & Blood pressure & Duration & Retinopathy /Retinopathy & Albuminuria |
| Kato, Takemori et al. 2002 [38] | Prospective study | 3614 | 5 years | Japan | Duration/Retinopathy |
| Hadjadj, Duly-Bouhanick et al. 2004 [39] | Prospective study | 297 | 7 years | France | HbA1c & Duration/Retinopathy |
| Tapp, Zimmet et al. 2006 [40] | Prospective study | 528 | 6 years | Australia | HbA1c & Duration/Retinopathy |
| Torffvit, Agardh et al. 1991 [41] | Cross Sectional | 476 | - | Sweden | Blood pressure & Albuminuria/Retinopathy & Albuminuria |
| Torffvit, Agardh et al. 1991 [42] | Cross Sectional | 451 | - | Sweden | HbA1c & Blood pressure & Duration/Albuminuria |
| Florkowski, Scott et al. 1998 [43] | Prospective study | 447 | 6 years | New Zealand | HbA1c & Blood pressure & Duration/Retinopathy |
| Tapp, Zimmet et al. 2006 [44] | Cross Sectional | 2182 | - | Australia | HbA1c/Retinopathy & Albuminuria |
| Izumi, Hoshi et al. 1995 [45] | Retrospective study | 107 | 3.2 years | Japan | HbA1c/Retinopathy |
| Parving 1998 [46] | Review | - | - | Finland | Albuminuria & Duration /Albuminuria |
| Raman, Verma et al. 2011 [47] | Prospective study | 1414 | 3 years | India | HbA1c/Retinopathy |
| Ohkubo, Kishikawa et al. 1995 [48] | Prospective study | 110 | 6 years | Japan | HbA1c/Retinopathy & Albuminuria |
| Ã–zmen and Boyvada 2003 [49] | Prospective study | 267 | 1 years | Turkey | HbA1c & Duration/Retinopathy |
| Lloyd, Klein et al. 1995 [50] | Retrospective study | 657 | 2 years | US | HbA1c/Retinopathy |
| Perol, Balkau et al. 2012 [51] | Retrospective study | 254 | 3 years | France | HbA1c & Albuminuria /Retinopathy |
| Kingsley, Dorman et al. 1988 [52] | Retrospective study | 754 | - | US | Duration/Retinopathy |
| Sasaki, Horiuchi et al. 1990 [53] | Prospective study | 976 | 8.3 years | Japan | HbA1c & Duration/Retinopathy |
| Voutilainen-Kaunisto, TerÃ¤svirta et al. 2000 [54] | Prospective study | 277 | 10 years | Finland | HbA1c & Duration/Retinopathy |
| Wan Nazaimoon, Letchuman et al. 1999 [55] | Cross Sectional | 926 | - | Malaysia | Blood pressure & Duration /Retinopathy & Albuminuria |
| Lu, Wen et al. 2007 [56] | Cross sectional | 1039 | -- | China | HbA1c & Blood pressure & Duration & Albuminuria/Retinopathy & Albuminuria |
| Raile, Galler et al. 2007 [57] | Prospective study | 27805 | 2.5 years | Germany | HbA1c & Blood pressure & Duration & Albuminuria/Albuminuria |
| Esmatjes and De Alvaro 2002 [58] | Prospective study | 1225 | 4.3 years | Spain | HbA1c & Duration/ Albuminuria |
| Garancini, Gallus et al. 1988 [59] | Cross sectional | 1155 | - | Italy | Duration/Albuminuria |
| Nomiyama, Nunoi et al. 1991 [60] | Cross sectional | 992 | - | Japan | HbA1c & Blood pressure & Duration /Albuminuria |
| Sasaki, Horiuchi et al. 1989 [61] | Retrospective study | 1196 | 10 years | Japan | HbA1c & Blood pressure & Duration & Retinopathy/Albuminuria |
| Bleyer, Sedor et al. 2008 [62] | Cross sectional | 296 |  | US | HbA1c & Blood pressure & Duration /Albuminuria |
| Agardh, Agardh et al. 1997 [63] | Prospective study | 442 | 5 years | Sweden | HbA1c & Albuminuria & Blood pressure /Albuminuria & Retinopathy |
| Kalk, Osler et al. 1990 [64] | Cross sectional | 127 |  | S. Africa | HbA1c & Blood pressure & Duration /Albuminuria |
| Giordano, Amato et al. 2011 [65] | Retrospective study | 376 | 10.93 years | Italy | HbA1c & Duration /Albuminuria & Retinopathy |
| Lunetta, M., et al. 1998 [66] | Cross sectional | 320 | - | Italy | Blood pressure & Retinopathy/Albuminuria |
| Miki, E. and M. Kikuchi 1994 [67] | Retrospective study | 2000 | 28 | Japan | HbA1c & Blood pressure & Duration/Retinopathy |

**REFERENCES**

References

1. Brownlee M, Aiello L P, Cooper M E, Vinik A I, Nesto R W, Boulton A J M (2011) Complications of diabetes mellitus. Williams textbook of endocrinology / shlomo melmed [et al]. 12th ed. Philadelphia: Elsevier/Saunders.

2. DCCT (1993) The effect of intensive treatment of diabetes on the development and progression of long-term complications in insulin-dependent diabetes mellitus. New England Journal of Medicine 329: 977.

3. Klein R, Klein B E, Moss S E, Davis M D, DeMets D L (1984) The wisconsin epidemiologic study of diabetic retinopathy. Ii. Prevalence and risk of diabetic retinopathy when age at diagnosis is less than 30 years. Arch Ophthalmol 102: 520.

4. DCCT (2000) Retinopathy and nephropathy in patients with type 1 diabetes four years after a trial of intensive therapy. New England Journal of Medicine 342: 381.

5. DCCT (1995) The relationship of glycemic exposure (hba1c) to the risk of development and progression of retinopathy in the diabetes control and complications trial. Diabetes 44: 968.

6. UKPDS (1998) Intensive blood-glucose control with sulphonylureas or insulin compared with conventional treatment and risk of complications in patients with type 2 diabetes (ukpds 33). The Lancet 352: 837(17).

7. Chase H P, Jackson W E, Hoops S L, Cockerham R S, Archer P G, O'Brien D (1989) Glucose control and the renal and retinal complications of insulin-dependent diabetes. JAMA, The Journal of the American Medical Association 261: p1155(6).

8. Krolewski A S, Canessa M, Warram J H, Laffel L M, Christlieb A R, Knowler W C, et al. (1988) Predisposition to hypertension and susceptibility to renal disease in insulin-dependent diabetes mellitus. N Engl J Med 318: 140.

9. Krolewski A S, Warram J H, Rand L I, Christlieb A R, Busick E J, Kahn C R (1986) Risk of proliferative diabetic retinopathy in juvenile-onset type i diabetes: A 40-yr follow-up study. Diabetes Care 9: 443.

10. Klein B E, Moss S E, Klein R (1990) Is menarche associated with diabetic retinopathy? Diabetes Care 13: 1034.

11. Kostraba J N, Klein R, Dorman J S, Becker D J, Drash A L, Maser R E, et al. (1991) The epidemiology of diabetes complications study. Iv. Correlates of diabetic background and proliferative retinopathy. Am J Epidemiol 133: 381.

12. Klein B E, Moss S E, Klein R (1990) Effect of pregnancy on progression of diabetic retinopathy. Diabetes Care 13: 34.

13. Al-Futaisi A, Al-Zakwani I, Almahrezi A, Al-Hajri R, Al-Hashmi L, Al-Muniri A, et al. (2006) Prevalence and predictors of microalbuminuria in patients with type 2 diabetes mellitus: A cross-sectional observational study in oman. Diabetes Research and Clinical Practice 72: 212.

14. Thai Multicenter Research Group on Diabetes Mellitus (1994) Vascular complications in non-insulin dependent diabetics in thailand. Diabetes Research and Clinical Practice 25: 61.

15. UKPDS (1998) Tight blood pressure control and risk of macrovascular and microvascular complications in type 2 diabetes: Ukpds 38. Uk prospective diabetes study group. BMJ 317: 703.

16. Kullberg C E, Arnqvist H J (1993) Elevated long-term glycated haemoglobin precedes proliferative retinopathy and nephropathy in type 1 (insulin-dependent) diabetic patients. Diabetologia 36: 961.

17. Krolewski A S, Laffel L M B, Krolewski M, Quinn M, Warram J H (1995) Glycosylated hemoglobin and the risk of microalbuminuria in patients with insulin-dependent diabetes mellitus. New England Journal of Medicine 332: 1251.

18. Savage S, Estacio R O, Jeffers B, Schrier R W (1996) Urinary albumin excretion as a predictor of diabetic retinopathy, neuropathy, and cardiovasculary disease in niddm. Diabetes Care 19: 1243.

19. Fujisawa T, Ikegami H, Yamato E, Kawaguchi Y, Ueda H, Shintani M, et al. (1999) Association of plasma fibrinogen level and blood pressure with diabetic retinopathy, and renal complications associated with proliferative diabetic retinopathy, in type 2 diabetes mellitus. Diabetic Medicine 16: 522.

20. Mogensen C E, Christensen C K (1984) Predicting diabetic nephropathy in insulin-dependent patients. N Engl J Med 311: 89.

21. Bruce A P, Linda H F, Kristen H S, Dianne M F, et al. (2003) Regression of microalbuminuria in type 1 diabetes. The New England Journal of Medicine 348: 2285.

22. Hovind P, Tarnow L, Rossing P, Jensen B R, Graae M, Torp I, et al. (2004) Predictors for the development of microalbuminuria and macroalbuminuria in patients with type 1 diabetes: Inception cohort study. BMJ 328: 1105.

23. Mogensen C E (1982) Long-term antihypertensive treatment inhibiting progression of diabetic nephropathy. BMJ 285: 685.

24. Dorsey R R (2006) Screening for chronic complications in type 1 diabetes [Ph.D.]. United States -- Pennsylvania: University of Pittsburgh.

25. DCCT (1996) The absence of a glycemic threshold for the development of long-term complications: The perspective of the diabetes control and complications trial. (the diabetes control and complications trial research group). Diabetes 45: 1289(10).

26. Gæde P, Vedel P, Larsen N, Jensen G V H, Parving H-H, Pedersen O (2003) Multifactorial intervention and cardiovascular disease in patients with type 2 diabetes. New England Journal of Medicine 348: 383.

27. Nathan D M, Cleary P A, Backlund J Y, Genuth S M, Lachin J M, Orchard T J, et al. (2005) Intensive diabetes treatment and cardiovascular disease in patients with type 1 diabetes. The New England Journal of Medicine 353: 2643.

28. Olsen B S, SjÃ¸lie A-K, Hougaard P, Johannesen J, Borch-Johnsen K, Marinelli K, et al. (2000) A 6-year nationwide cohort study of glycaemic control in young people with type 1 diabetes: Risk markers for the development of retinopathy, nephropathy and neuropathy. Journal of Diabetes and its Complications 14: 295.

29. Romero-Aroca P, Baget-Bernaldiz M, Fernandez-Ballart J, Plana-Gil N, Soler-Lluis N, Mendez-Marin I, et al. (2011) Ten-year incidence of diabetic retinopathy and macular edema. Risk factors in a sample of people with type 1 diabetes. Diabetes Research and Clinical Practice 94: 126.

30. Okudaira M, Yokoyama H, Otani T, Uchigata Y, Iwamoto Y (2000) Slightly elevated blood pressure as well as poor metabolic control are risk factors for the progression of retinopathy in early-onset japanese type 2 diabetes. Journal of Diabetes and its Complications 14: 281.

31. Golbahar J, Rahimi M, Tabei M B, Aminzadeh M A (2008) Clinical risk factors and association of hyperhomocysteinemia with diabetic retinopathy in iranian type 2 diabetes patients: A cross-sectional study from shiraz, southern iran. Diabetes and Metabolic Syndrome: Clinical Research and Reviews 2: 192.

32. Imano E, Miyatsuka T, Motomura M, Kanda T, Matsuhisa M, Kajimoto Y, et al. (2001) Heart rate elevation and diabetic retinopathy in patients with type 2 diabetes mellitus and normoalbuminuria. Diabetes Research and Clinical Practice 52: 185.

33. Tam V H K, Lam E P K, Chu B C Y, Tse K K, Fung L M (2009) Incidence and progression of diabetic retinopathy in hong kong chinese with type 2 diabetes mellitus. Journal of Diabetes and its Complications 23: 185.

34. Semeraro F, Parrinello G, Cancarini A, Pasquini L, Zarra E, Cimino A, et al. (2011) Predicting the risk of diabetic retinopathy in type 2 diabetic patients. Journal of Diabetes and its Complications 25: 292.

35. Massin P, Erginay A, Mercat-Caudal I, Vol S, Robert N, Reach G, et al. (2007) Prevalence of diabetic retinopathy in children and adolescents with type-1 diabetes attending summer camps in france. Diabetes &amp; Metabolism 33: 284.

36. Rema M, Ponnaiya M, Mohan V (1996) Prevalence of retinopathy in non insulin dependent diabetes mellitus at a diabetes centre in southern india. Diabetes Research and Clinical Practice 34: 29.

37. Romero P, Salvat M, FernÃ¡ndez J, Baget M, Martinez I (2007) Renal and retinal microangiopathy after 15 years of follow-up study in a sample of type 1 diabetes mellitus patients. Journal of Diabetes and its Complications 21: 93.

38. Kato S, Takemori M, Kitano S, Hori S, Fukushima H, Numaga J, et al. (2002) Retinopathy in older patients with diabetes mellitus. Diabetes Research and Clinical Practice 58: 187.

39. Hadjadj S, Duly-Bouhanick B, Bekherraz A, Bridoux F, Gallois Y, Mauco G, et al. (2004) Serum triglycerides are a predictive factor for the development and the progression of renal and retinal complications in patients with type 1 diabetes. Diabetes &amp; Metabolism 30: 43.

40. Tapp R J, Zimmet P Z, Harper C A, McCarty D J, Chitson P, Tonkin A M, et al. (2006) Six year incidence and progression of diabetic retinopathy: Results from the mauritius diabetes complication study. Diabetes Research and Clinical Practice 73: 298.

41. Torffvit O, Agardh E, Agardh C D (1991) Albuminuria and associated medical risk factors: A cross-sectional study in 476 type i (insulin-dependent) diabetic patients. Part 1. J Diabet Complications 5: 23.

42. Torffvit O, Agardh E, Agardh C D (1991) Albuminuria and associated medical risk factors: A cross-sectional study in 451 type ii (noninsulin-dependent) diabetic patients. Part 2. Journal of Diabetic Complications 5: 29.

43. Florkowski C M, Scott R S, Moir C L, Graham P J (1998) Clinical and biochemical outcomes of type 2 diabetes mellitus in canterbury, new zealand: A 6-year cohort study. Diabetes Research and Clinical Practice 40: 167.

44. Tapp R J, Zimmet P Z, Harper C A, de Courten M P, McCarty D J, Balkau B, et al. (2006) Diagnostic thresholds for diabetes: The association of retinopathy and albuminuria with glycaemia. Diabetes Research and Clinical Practice 73: 315.

45. Izumi K, Hoshi M, Kuno S, Okuno G, Yamazaki Y, Isshiki G, et al. (1995) Glycemic control, growth and complications in children with insulin-dependent diabetes mellitus - a study of children enrolled in a summer camp program for diabetics in kinki district, japan. Diabetes Research and Clinical Practice 28: 185.

46. Parving H H (1998) Renoprotection in diabetes: Genetic and non-genetic risk factors and treatment. Diabetologia 41: 745.

47. Raman R, Verma A, Pal S S, Gupta A, Vaitheeswaran K, Sharma T (2011) Influence of glycosylated hemoglobin on sight-threatening diabetic retinopathy: A population-based study. Diabetes Research and Clinical Practice 92: 168.

48. Ohkubo Y, Kishikawa H, Araki E, Miyata T, Isami S, Motoyoshi S, et al. (1995) Intensive insulin therapy prevents the progression of diabetic microvascular complications in japanese patients with non-insulin-dependent diabetes mellitus: A randomized prospective 6-year study. Diabetes Research and Clinical Practice 28: 103.

49. Ã–zmen B, Boyvada S (2003) The relationship between self-monitoring of blood glucose control and glycosylated haemoglobin in patients with type 2 diabetes with and without diabetic retinopathy. Journal of Diabetes and its Complications 17: 128.

50. Lloyd C E, Klein R, Maser R E, Kuller L H, Becker D J, Orchard T J (1995) The progression of retinopathy over 2 years: The pittsburgh epidemiology of diabetes complications (edc) study. Journal of Diabetes and its Complications 9: 140.

51. Perol J, Balkau B, Guillausseau P J, Massin P (2012) A study of the 3-year incidence of diabetic retinopathy in a french diabetic population seen at lariboisiere hospital, paris. Diabetes &amp; Metabolism.

52. Kingsley L A, Dorman J S, Doft B H, Orchard T J, LaPorte R E, Kuller L H, et al. (1988) An epidemiologic approach to the study of retinopathy: The pittsburgh diabetic morbidity and retinopathy studies. Diabetes Research and Clinical Practice 4: 99.

53. Sasaki A, Horiuchi N, Hasewgawa K, Uehara M (1990) Development of diabetic retinopathy and its associated risk factors in type 2 diabetic patients in osaka district, japan: A long-term prospective study. Diabetes Research and Clinical Practice 10: 257.

54. Voutilainen-Kaunisto R M, TerÃ¤svirta M E, Uusitupa M I J, Niskanen L K (2000) Occurrence and predictors of retinopathy and visual acuity in type 2 diabetic patients and control subjects: 10-year follow-up from the diagnosis. Journal of Diabetes and its Complications 15: 24.

55. Wan Nazaimoon W M, Letchuman R, Noraini N, Ropilah A R, Zainal M, Ismail I S, et al. (1999) Systolic hypertension and duration of diabetes mellitus are important determinants of retinopathy and microalbuminuria in young diabetics. Diabetes Research and Clinical Practice 46: 213.

56. Lu B, Wen J, Song X Y, Dong X H, Yang Y H, Zhang Z Y, et al. (2007) High prevalence of albuminuria in population-based patients diagnosed with type 2 diabetes in the shanghai downtown. Diabetes Research and Clinical Practice 75: 184.

57. Raile K, Galler A, Hofer S, Herbst A, Dunstheimer D, Busch P, et al. (2007) Diabetic nephropathy in 27,805 children, adolescents, and adults with type 1 diabetes. Diabetes Care 30: 2523.

58. Esmatjes E, De Alvaro F (2002) Incidence of diabetic nephropathy in type 1 diabetic patients in spain: Estudio diamante. Diabetes Research and Clinical Practice 57: 35.

59. Garancini P, Gallus G, calori G, Micossi P, Pozza G (1988) Microalbuminuria and its associated risk factors in a representative sample of italian type ii diabetics. Journal of Diabetic Complications 2: 12.

60. Nomiyama R, Nunoi K, Tsutsu N, Satho Y, Yoshizumi H, Himeno H, et al. (1991) One-day survey of albuminuria in diabetic outpatients in fukuoka prefecture, japan. Journal of Diabetic Complications 5: 155.

61. Sasaki A, Horiuchi N, Hasagawa K, Uehara M (1989) Persistent albuminuria as an index of diabetic nephropathy in type 2 diabetic patients in osaka, japan. Incidence, risk factors, prognosis and causes of death. Diabetes Research and Clinical Practice 7: 299.

62. Bleyer A J, Sedor J R, Freedman B I, O'Brien A, Russell G B, Graley J, et al. (2008) Risk factors for development and progression of diabetic kidney disease and treatment patterns among diabetic siblings of patients with diabetic kidney disease. American Journal of Kidney Diseases 51: 29.

63. Agardh C D, Agardh E, Torffvit O (1997) The association between retinopathy, nephropathy, cardiovascular disease and long-term metabolic control in type 1 diabetes mellitus: A 5 year follow-up study of 442 adult patients in routine care. Diabetes Research and Clinical Practice 35: 113.

64. Kalk W J, Osler C, Taylor D, Panz V R, Esse J D, Reinach S G (1990) The prevalence of micro-albuminuria and glomerular hyperfiltration in young patients with iddm. Diabetes Research and Clinical Practice 8: 145.

65. Giordano C, Amato M C, Ciresi A, Citarrella R, Mantione L, Accidenti M, et al. (2011) Predictors of microvascular complications in type 1 diabetic patients at onset: The role of metabolic memory. European Journal of Internal Medicine 22: 266.

66. Lunetta M, Infantone L, Calogero A E, Infantone E (1998) Increased urinary albumin excretion is a marker of risk for retinopathy and coronary heart disease in patients with type 2 diabetes mellitus. Diabetes Research and Clinical Practice 40: 45.

67. Miki E, Kikuchi M (1994) Diabetic retinopathy and control of diabetes with special reference to blood glucose levels. Diabetes Research and Clinical Practice 24, Supplement: S177.
