# Supplementary material for: Applying a Novel Combination of Techniques to Develop a Predictive Model for Diabetes Complications
Source: PLoS One. 2015 Apr 22;10(4):e0121569. doi: 10.1371/journal.pone.0121569 (PMC4406519; doi:10.1371/journal.pone.0121569)
Supplement: S3 File — (DOCX) [file pone.0121569.s003.docx]

**Supplementary File 3**

**
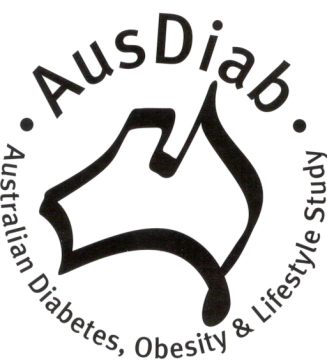
**

**ACCESS TO DATA, BIOLOGICAL MATERIALS AND SUBJECTS**

The AusDiab team welcomes approaches from bona fide research workers for access to the accumulated data and biological materials and for participation in ongoing and new data collection activities.

1. Access will generally not be granted to research groups/institutions or individuals who are receiving support from the Australian Tobacco Research Foundation or other bodies associated with the tobacco industry.

**Investigators may apply for access to**

1. information on survey subjects from the computerised database,
2. the accumulated biological materials (blood sera ) on survey subjects in the specimen storage facility,
3. the survey subjects for the purpose of collecting additional information,
4. participation in new AusDiab data collection activities.

**How to apply**

1. Applications must be in writing and in accordance with the guidelines below. **Lengthy submissions are not usually required.** Data and biological materials will not be provided for approved projects until evidence is provided that the appropriate Institutional Ethics Committee (IEC) approval has been granted, and the responsible investigators have undertaken in writing to abide by stated conditions (see Undertakings by investigators).

All applications are considered by the AusDiab Scientific Research Committee and, in general, access will be granted if

1. Serious scientific research is proposed that is consistent with the overall program of research activities;
2. Research proposed does not conflict with work in progress;
3. The interests and personal privacy of survey subjects are protected and Institutional Ethics Committee approval has been given for the proposed research;
4. Resources are available to pay the AusDiab and associated service providers for the data and materials and for the work to be done in making the data and biological materials available, and also for the further work proposed by the investigator;
5. The responsible investigator agrees to provide a copy of any additional data collected on survey subjects to AusDiab for possible later use by other researchers.

**Types of access**

For applications limited to **(a)** and **(b)** above, data may be made available in the form of completed statistical analyses, data summaries such as cross-tabulations, or a de-identified unit-record data file. Biological materials may be made available in the form of sera or DNA specimens, or new analyses on stored specimens may be arranged in collaboration with AusDiab-affiliated personnel.

For applications involving **(c)** above, name-identified data may be provided or arrangements will be made for AusDiab-affiliated personnel to collect the additional data on behalf of the investigators.

1. Applications involving **(d)** must be compatible with the on-going program of research activities and the responsible investigator is usually required to become a member of the AusDiab team.

**Access fees**

There are two fees. The first is based on full-cost recovery for the programming and other costs associated with the planning, extraction and provision of the data. The second is a contribution to the on-going storage, maintenance and other infrastructure costs of the AusDiab, both are payable to IDI / AusDiab – epidemiology unit.

The latter fee depends upon the type and amount of data and biological materials required, the association of the investigators with the AusDiab program of activities and the intended use of the data.

Investigators may, prior to submission of the formal application, submit a brief expression of interest and request a list of available variables / including a brief description in order to ascertain the suitability of the available data and materials for their research aims.

Publication of reports arising from successful applications – see appendix A for further information.

**Guidelines for the preparation of applications**

The following headings should be used in the application. In addition, please indicate whether the aim is for publication of findings, or just for internal analysis.

1. **Responsible Investigators**
   Name, degrees and professional qualifications, title of appointment held, department and institution of each responsible investigator.
2. **Proposed Research**
   1. Title of project.
   2. Background and rationale
   3. Specific aims
   4. Research plan
3. **Data and biological materials**
   1. Supply full details of data variables and biological materials required
   2. Specify the form in which the data and biological materials are required
4. **Resources**
   1. What services are requested by AusDiab in provision or analysis of data and biological materials and what funds are available to pay for these services.
   2. What funds or other resources are available to the investigator to undertake the proposed work.
5. **Ethical Considerations**
   1. If name-identified data are requested, describe the provisions that will be made to protect their confidentiality.
   2. If subjects are to be approached in any way, give full details of proposed contact and data collection.
   3. Provide evidence (at the time of submission or later) that the proposed research project has been approved by an institutional ethics committee
6. **Undertakings and signatures**
   Complete and sign Undertakings by responsible investigators.

**Applications must be submitted to:**

Associate Professor Jonathan Shaw
International Diabetes Institute,

250 Kooyong Road,

Caulfield South Vic, Australia 3162

**Undertakings by responsible investigators**

**Title of project**

|  |
| --- |

**Responsible investigators**

| Name  Institution  Department | Name  Institution  Department |
| --- | --- |
| Name  Institution  Department | Name  Institution  Department |

**Institution where project will be carried out**

**Name of institution**

|  |
| --- |

**Head of department/section**

| Name | Signature |
| --- | --- |

**Undertakings by investigators**

The responsible investigators undertake to:

1. Protect the interests and privacy of survey subjects;
2. Provide evidence of institutional ethics committee approval of project;
3. Pay all required fees in a timely manner;
4. Limit the research to that proposed in the original application (unless a further submission is made and approved);
5. Provide AusDiab with a copy of all additional data collected on subjects;
6. Provide AusDiab with a report on the outcome of the research;
7. Provide to AusDiab a copy of any proposed publication for comment before its submission for publication;
8. Return unused biological materials to the Institute immediately as soon as the project is completed;
9. AusDiab should be mentioned in the title and/or abstract of all publications using AusDiab data and materials;
10. Acknowledge the International Diabetes Institute and the AusDiab community in the manner specified by the International Diabetes Institute, in any report or publication about the research;

**Signatures of investigators**

| Name | Signature | Date |
| --- | --- | --- |
| Name | Signature | Date |
| Name | Signature | Date |
| Name | Signature | Date |

1. **Appendix A: Publication Policy**

All publications arising from access to AusDiab data will be reviewed by the Director of Epidemiology – International Diabetes Institute (IDI) or his nominated representative in accordance with the following guidelines.

1. Data Analysis and Release of Results

The scientific integrity of the project requires that the data from all State and Territory sites be analysed study-wide and reported as such. Thus an individual State or Territory is not expected to report on the data collected from their region alone. The development of reports on data from individual States and Territories is the prerogative of the International Diabetes Institute.

All presentations and publications are expected to protect the integrity of the major objective(s) of the study and maintain confidentiality of participants.

1. Review Process

Each paper or abstract, as described below, must be submitted to the AusDiab Steering Committee through IDI, for review of its appropriateness and scientific merit at least six weeks prior to submission. The AusDiab Steering Committee may recommend changes prior to approval. The final decision regarding content, presentation and interpretation of the results contained in the manuscripts is that of the AusDiab Steering Committee.

1. Primary Outcome Papers

The primary outcome papers of the AusDiab study are papers that present data on the development of DM in the AusDiab participants. The determination of whether or not a particular paper represents a primary outcome paper will be made by the AusDiab Steering Committee.

Primary outcome papers will not have named individual authors but will be published under the by-line of the AusDiab Principal Investigators.

1. Other Study Papers, Abstracts and Presentations

All studies other than those designated as “Primary Outcome” fall in this category. Papers or abstracts resulting from these studies will have named authorship of individuals involved, ending with the phrase “on behalf of the AusDiab Steering Committee”. In addition, papers will have an appendix containing the names of Collaborating Centres and Principal Investigators.
